# Observations and Reflections Relative to the Treatment of Burns

**Published:** 1819-05

**Authors:** Hannah Barnard, Samuel L. Mitchel

**Affiliations:** Hudson, in the United States; New-York.


*For the London Medical and Physical Journal.*

### *Observations and Reflections relative to the Treatment of Burns;* by Mrs. Hannah Barnard, of Hudson, in the United States: *with some Remarks on them,* by Dr. Samuel L. Mitchel, of New-York.*

*To Dr. Samuel L. Mitchel.*

*Hudson; Oct.* 6, 1818.

THE only apology I judge necessary for the freedom of this address is, that I believe I am writing to a gentleman, a physician, a philosopher, and philanthropist. I therefore take the liberty of stating to thee a discovery which I accidentally made about thirteen years ago, in which, natural philosophy had little or no agency, (whatever degree of it I might flatter myself with having previously acquired,) but by enabling me to trace back to the cause the unexpected and surprising effect.

---

*** We insert this article amongst our *national* Original Communications, in consequence of its not being strictly conformable in character to that which we desire the subjects comprised in our *Foreign Department* should possess. The space in our Journal required for the insertion of the observations of the enlightened Mrs. Hannah Barnard will, we consider, be well occupied: we only think it more proper that that space should be assigned from the part of it which is devoted to miscellaneous subjects.**

I had burnt the back of my thumb, near the hand, a space perhaps less than the size of a dollar, which was nevertheless sufficient "to tie down my sore attention" to its smarting for two or three hours, while busily engaged in domestic avocations. At length, merely because I knew not what to do with it, I applied a plaster, compounded of Burgundy pitch, bees'-wax, and a little oil; which I had long kept in the house as a convenient application for slight wounds, and which I shall take the liberty hereafter more particularly to describe. I then went on with my work, and did not think of my burn again till about five hours after, when the singular circumstance of such complete relief excited an immediate investigation of the cause; which, as it appeared to me, was, first, the component parts of the natural covering, the skin, were so far decomposed or weakened by the action of fire as to render them incapable of bearing the application of oxygen to that part, without suffering a continued tendency to further dissolution, that the external application of a complete non-conductor gave the part immediate rest, and afforded nature an opportunity to repair the breach. By thus excluding the brisk action of oxygen, every tendency to inflammation from without was also fully excluded.

In consequence of the conviction resulting from this train of reasoning, I have never since made any other application to a burn or scald; and, by a continued series of invariably-successful trials, I am so fully confirmed in the rationality of the theory, that I now feel it an incumbent duty I owe to suffering humanity, the infant part of it especially, to use every effort in my power to give it publicity. And, though I flatter myself it will not be necessary to corroborate it by facts in order to obtain Dr. Mitchell's assent to the justness of my theory, yet it may be to some others, to whom thou mayst have the goodness to communicate it, from the same benevolent and compassionate motives which, I trust, have induced me to make this candid statement. I shall, therefore, now select three of the most prominent cases out of the many to which I have been witness, or which have been substantiated to me by what I consider unquestionable authority.

The first was the case of a young woman in our family, eight or nine years ago, who scalded her arm with a column of steam, which raised an entire blister on about one-third of its surface. I applied the plaster, and bound it up close: it gave her immediate and complete relief from any further suffering. She let it remain four or five days without opening, and pursued her work as usual. In little more than a week it was completely healed, and no inflammation ever appeared in it.

The second case was that of a child about a year old, in the summer of 1817, who was scalded with salt-meat broth on the breast and nearly the whole of the right arm. The father, whose name is Nichols, came nearly six miles to me for directions; having previously heard something about my mode of treating burns and scalds. This was the afternoon of first-day [Sunday]; and, before the week was out, he informed my late lamented brother-in-law, Richard Robotham, that, on the application of the plaster, the child went quietly to sleep, after suffering extremely during four or five hours; had a good night's rest; that the parts were nearly all healed; and the child had, through the whole process, been entirely easy and free from fever.

The third is a recent instance of its good effects in the case of a child of David Rogers in this town, about four years old, who was scalded on the 24th ult. We judged about one half the surface of the right leg was blistered; and, in the bend of the ankle, where the stocking was wrinkled, and held the heat longer, the flesh was destroyed under the skin, apparently more than the skin's thickness. The leg was immediately wrapped close in cotton, until the salve could be made and a plaster applied; which could not take less than three-quarters of an hour, during which time the child's suffering was extreme. In less than ten minutes after the plaster was on, she was perfectly easy, and in less than ten more was asleep; and has never since made the least complaint of smarting pain or soreness. Next morning the blisters were carefully pierced, on the under side, with a large needle, through the plaster and skin, when the water copiously flowed; after which the plaster was drawn a little closer and bandaged snug, but was not taken off till the third day, and then with great care not to break the skin, only with a large needle to let out the water, which had again accumulated. The leg was then, without washing, again enclosed in the plaster, after adding a little more salve where it appeared to be necessary. I attended it every day, merely for the sake of critically marking its progress; for the child had in its maternal grandmother one of the best of nurses, in whose skill and attention I placed the most entire confidence. About the fifth day there were plain indications of healing, by great part of the space ceasing to discharge. On the ninth the new skin was formed evidently over the whole. On the tenth the plaster was removed entirely, and the leg only wrapt in a cloth wetted with spirits, and a bandage applied, merely to shield the young skin from the air, and prevent the child's taking cold after having the limb so much wrapt up. This day, the eleventh from the accident, the leg appears wholly free from redness, or even tetters, so common on the healing of burns which have suffered in their progress by inflammation to any considerable degree; and it has never been swelled at all.

I now respectfully request thee to inform me on the following points:—first, to what extent may a non-conductor be closely applied to the superfices of the human body, and yet leave sufficient space for the necessary oxygenation of the blood, through that source, to preserve it in a healthy state?—also whether, and in what degree, oxygen can be artificially increased through the lungs, with safety to that important organ?

With regard to the composition, I would just observe, that, though the pitch and wax are, as thou knowest, equally non-conductors, yet the pitch alone, even when softened with oil, is more adhesive than is necessary; the wax not enough so. I, therefore, allow one quarter or a little more wax, with a little lard, fresh butter, or oil, to soften the composition sufficiently, but not so as to cause it to melt away with the warmth of the flesh and admit the air, which would destroy its effect as a non-conductor. I then spread it with a hot knife on old nankeen, or any other close limber cloth: leather is not so good, as, on any moisture getting to it, when it afterwards becomes dry, it is apt to grow hard. If the skin is rubbed off in any part, I first cover the part with a little soft linen lint, and then apply the plaster close, and bandage it carefully, to secure it from slipping. I trust thy goodness will readily pardon my thus attempting to light a candle at noon-day; and, with requesting a line from thee when leisure from more important avocations permits, I subscribe, with high consideration and esteem, thy assured friend, HANNAH BARNARD.

*To Mrs. Hannah Barnard.*

ON looking over my letters requiring answers this morning, I find yours to be one. I had very often heard of the writer as a person of extensive observation and enlarged mind: I now behold her as a physician and a philanthropist.

Pain is so great an evil, that I wish by every rational means to lessen its dominion. The facts stated in your communication are very interesting, and present the accidents of burns and scalds in a new aspect to me. Ice, cotton, spirit of turpentine, and various other remedies, were familiar to me; and, although I knew the employment of *cerate*, or a composition of bees-wax and olive-oil, for a dressing for wounds or sores, I did not know that it could assuage the torment consequent upon accidents by fire.

These casualties are so frequent and distressing, that it is important that an efficacious and ready method of treating them should be known to the head of every family, as also to every medical practitioner.

The theory of the manner in which the plaster acts is ingenious, and I will add so probable, that I am not acquainted with a better. It is certain that the oxygen of the atmosphere is in contact with the whole surface of our bodies: it penetrates the windpipe and lobes of the lungs; it passes into the alimentary canal; and in all these situations it has more than a mere mechanical operation. The other effect to which I allude is chemical:—when the human body, or any part of it, or any animated substance, is heated to such a temperature as to attract oxygen, a combustion, more or less rapid, takes place: they call it inflammation; and, in this view of the subject, the term is very appropriate. If it be intended to repress the further accumulation and action of the caloric, the oxygenous part of the atmosphere must be kept away. A close application of an ointment or cerate does this: the part is protected from the assault of oxygen and caloric, and the consequent pain and anguish cease.

The analogy with inanimate matter is very strict: a piece of wood, when highly charged with caloric, may be kept from actual burning or decomposition by being separated from the oxygen of the air. In that case the fire is smothered, or does not kindle.

Why may not the interpretation apply to both cases? It has as much nice similitude as most of the theories we possess.

Permit me to conclude by an assurance of my great regard and esteem. SAML. L. MITCHEL.

